# ERCC1 and MGMT Methylation as a Predictive Marker of Relapse and FOLFOX Response in Colorectal Cancer Patients from South Tunisia

**DOI:** 10.3390/genes14071467

**Published:** 2023-07-19

**Authors:** Dhouha Jamai, Raja Gargouri, Boulbaba Selmi, Abdelmajid Khabir

**Affiliations:** 1 Research Laboratory of Bioresources, Integrative Biology and Valorization LR14ES06, Higher Institute of Biotechnology of Monastir, University of Monastir, Avenue Tahar Haaadded, BP 74, Monastir 5000, Tunisia; 2 Department of Pathology, Habib Bourguiba University Hospital, Medenine 4100, Tunisia; 3 Laboratory of Molecular Biotechnology of Eukaryotes, Biotechnology Center, University of Sfax, Avenue Sidi Mansour, Sfax 3018, Tunisia

**Keywords:** colorectal cancer, MGMT, ERCC1, methylation, relapse, FOLFOX response

## Abstract

Genetic and epigenetic modifications present a major cause of relapse and treatment failure in colorectal cancer. This study aims to appreciate the prognostic and predictive value of ERRC1 and MGMT methylation. We also studied the prognostic impact of the ERCC1 rs11615 polymorphism as well as its expression. Methylation profiles of ERCC1 and MGMT were tested by methylation-specific PCR. A polymorphism of ERCC1 was studied using PCR-RFLP and its expression was examined by immunohistochemistry. ERCC1 was methylated in 44.6% of colorectal adenocarcinoma while MGMT was methylated in 69% of cases. MGMT methylation was strongly associated with lymph node metastasis, lymph invasion, venous invasion, perineural invasion, distant metastasis and relapse. Patients with methylation of both genes were more likely to have a poor prognosis and display chemoresistance. IHC analysis revealed that ERCC1 staining was noted in 52.8% of colorectal adenocarcinoma and inversely related to distant metastasis and cancer recurrence. Kaplan Meier analysis revealed that the worst overall survival was significantly associated with ERCC1 and MGMT methylation while decreased ERCC1 expression and T/T genotype exhibited the best overall survival. The methylation of MGMT, alone or combined with ERCC1, is predictive for poor prognosis, short overall survival and chemotherapy response in colorectal cancer.

## 1. Introduction

DNA repair plays an important role in maintaining genome integrity in both normal and tumor cells. Defects in DNA repair damage are a major source of potentially genetic alterations leading to carcinogenesis [1]. Although gene mutation occurs early in colorectal cancer, aberrant methylation of repair DNA gene is more frequent [2]. Understanding the epigenetic methylation is an urgent need to identify prognostic and predictive biomarkers of chemotherapy response. Currently, the expression of DNA repair genes such as the enzyme O-6-methylguanine-DNA methyltransferase (*MGMT*) and Excision Repair Cross-Complementation Group 1 (ERCC1) plays a potential role in colorectal cancer progression [3,4].

Excision Repair Cross-Complementation Group 1 (ERCC1) is a key protein in NER pathway. It is involved in repair DNA lesions caused by UV light or formed by chemotherapeutic agents [5,6,7]. Several studies appreciated its prognostic and predictive value. Hence, Jiang et al. showed that ERCC1 expression in cancer tissues was significantly higher than that in the adjacent tissues. Also, the low expression of this factor was closely related to a higher 3-year survival rate with good predictive value [8]. Instead, the study of Gajjar and his collaborators did not find any prognostic significance of ERCC1 expression [9]. Genetic as well as epigenetic changes in ERCC1 exhibited the major cause of chemoresistance. The single-nucleotide polymorphism ERCC1 C118T leads to a reduction in protein expression and alters DNA repair capacity. Hence, Rao1 et al. reported that progression-free survival was significantly lower with C/C or T/C compared to T/T [10]. Others demonstrated that better disease-free survival (DFS) was observed for ERCC1 (C/T + T/T) versus (C/C) [11]. Recently, case-control studies have investigated the role of ERCC1 polymorphisms in susceptibility to CRC. Salimzadeh et al. reported that The ERCC1-rs11615 variant was not linked to colorectal cancer risk while it was associated with chemotherapy toxicity [12]. Furthermore, a Chinese study confirmed that ERCC1 rs3212986 and rs2298881 polymorphisms increased CRC susceptibility while no association was found between ERCC1 polymorphisms rs11615 and colorectal cancer risk [13]. So far, there have been only limited studies focused on ERCC1 methylation in colorectal cancer. Shalaby et al. demonstrated that a high ERCC1 methylation rate was found in rectum cancer [14]. 

The DNA repair enzyme called O-6-methylguanine-DNA methyltransferase (*MGMT*) plays a crucial role in protecting against DNA damage caused by alkylating agents. It removes the alkyl group from the O^6^ position of guanine to protect cells from carcinogens. This renders it a chemoresistance biomarker. Hence, cancer cells with high MGMT levels might be resistant to alkylating chemotherapy [3,15]. MGMT methylation has not only predictive but also prognostic value. Therefore, CRC patients with MGMT promoter methylation showed a prolonged survival time [16]. Further, Li et al. demonstrated that MGMT promoter methylation represented a central event in the colorectal adenoma–carcinoma progression. However, no significant correlation was recorded between methylation status and the overall survival of CRC patients [17]. There are several reports on the methylation profile of ERCC1 and MGMT in colorectal cancer patients from different populations. To our knowledge, the methylation status of these genes in Tunisian colorectal cancer population is still not well-studied. Considering all these things, we found it rational to investigate the correlation between the methylation status of ERCC1 and MGMT, prognosis, survival and therapeutic response in colorectal cancer patients.

## 2. Materials and Methods

### 2.1. Patients and Samples

Our study population consisted of 111 colorectal cancer patients from south Tunisia between 2015 and 2020. Archival formalin-fixed and paraffin-embedded tumor and adjacent normal tissues from each patient were collected from the Department of Pathology, Habib Bourguiba University Hospital, Medenine, Tunisia, Department of Pathology, Habib Bourguiba University Hospital, Sfax, Tunisia and Laboratory of Pathology of Djerba, Tunisia. Samples were from 46 women and 65 men. Follow-up data were collected for each case by making phone calls and checking medical records. Follow-up ended on December 2021. The primary endpoints that we included were overall survival (OS). OS was calculated as the number of months from surgical operation until the follow-up deadline or the date of death.

This study was approved by the ethics committee of the High Institute of Biotechnology of Monastir, Tunisia. 

### 2.2. Immunohistochemistry

Immunohistochemistry technique was performed using the indirect avidin–biotin–peroxidase method to detect ERCC1 in tumor tissues. Paraffin-embedded TMA and tissue sections were deparaffinised and rehydrated in ethanol baths of decreasing degree. Slides were subjected to antigen retrieval (pH 9.0), followed by incubation in H_2_O_2_ to block endogenous peroxidase. Sections were washed in PBS and then incubated in β blocking to avoid non-specific binding. The primary antibody was applied on the sections with the appropriately diluted primary antibody (ERCC1 Monoclonal Antibody (8F1), Invitrogen, Waltham, MA, USA) (1/100). After PBS washing, slides were incubated for 25 min with the secondary antibody then with post-primary antibody. After the revelation with 3.3′-diaminobenzidine and the hematoxylin counter-staining, the sections were dehydrated and then mounted. 

### 2.3. DNA Extraction, Bisulfite Treatment, and Methylation-Specific PCR

Genomic DNA was extracted from tumor and normal tissue using the phenol-chloroform method. The purity and quantity of extracted DNA were checked using a NanoDrop ND-1000 spectrophotometer. DNA bisulfite treatment was carried out by EZ DNA Methylation Kit (Zymo Research, Irvine, CA, USA) according to the manufacturer’s protocol. Next, methylation-specific PCR (MSP) amplification was performed in a 50 μL volume reaction system that consisted of 50 ng sodium bisulfite-treated DNA, 200 ng of each primer, 1× PCR buffer, 2.5 mM dNTP and 1 U Taq polymerase (dream taq). 

Amplification was carried out with two different primer pairs specific to both methylated and unmethylated MGMT and ERCC1 promoter sequence. The primer sequences, annealing temperature specific to each primer, and size of PCR products are described in Table 1. 

The complete MSP conditions were as follows: 94 °C for 5 min, followed by 40 cycles of 94 °C for 30 s, Annealing temperature for 30 s, and 72 °C for 45 s, with a final extension at 72 °C for 7 min. Following amplification, the PCR products were separated on a 3% agarose gel containing ethidium bromide and visualized under UV illumination. 

### 2.4. ERCC1 Polymorphism Study by PCR-RFLP

The amplification of ERCC 118C->T polymorphism was performed using PCR-restriction fragment length polymorphism (PCR-RFLP). The polymorphic site 118C->T was amplified using the specific oligonucleotides shown in Table 1. PCR products were produced from 50 ng tumor DNA in a 25 μL final volume containing 1× PCR buffer, 200 μmol/L dNTPs, 0.4 μmol/L each primer, and 1U Taq polymerase (Invitrogen, Paisley, UK). The complete conditions of the amplification were as follows: 94 °C for 7 min, followed by 35 cycles of 94 °C for 30 s, 58 °C for 30 s, and 72 °C for 45 s, with a final extension at 72 °C for 10 min. 

PCR products were digested overnight at 37 °C with 1U of the enzyme BsrDI and then separated on 2% agarose gel containing ethidium bromide. Following digestion, the C/C genotype generated a single fragment of 208 pb, the 118 T allele produced two fragments of 128 bp and 80 bp and C/T genotype generated three fragments of 208 pb, 128 pb and 80 pb. 

### 2.5. Statistical Analysis

This study used SPSS program (version IBM SPSS Statistics 22) to perform statistical analysis on experimental data. Descriptive statistics were used to summarize clinicopathologic characteristics. Spearman analysis was used to evaluate the correlation between clinicopathological factors and proteins expression. Binary logistic regression analysis was performed to predict the impact of clinicopathological factors on the dependent variable (simultaneous methylation of ERCC1 and MGMT). Survival analysis was conducted using the Kaplan–Meier method and survival curves were compared using the Log-rank test. The accepted level of significance was *p* ≤ 0.05.

## 3. Results

### 3.1. Patients

One hundred and eleven patients diagnosed with colorectal adenocarcinoma were included in this study. Sixty-five patients (58.6%) were men and forty-six (41.4%) were women. The mean age of study population was 63 ± 14.40 years (65.40 ± 13.56 years for men and 59.53 ± 15.02 years for women). As shown in Table 2, about 68.5% of the patients in our study cohort were older than 50 years at the time of diagnosis. Tumors were located colon in 96 patients (86.5%) and rectum in 16 patients (13.5%), and all of them were diagnosed as having adenocarcinoma. According to the stratification of the 7th version of the American Joint Committee on Cancer (AJCC), 53.7% of the tumor belonged to T1–T2, and 46.3% belonged to T3–T4. Most of the lymph node statuses were positive (59.5%). About half of patients were diagnosed with metastatic colorectal cancer. A total of 27 cases received FOLFOX chemotherapy. The clinicopathological parameters are summarized in Table 2. 

### 3.2. Immunohistochemistry

**Figure 1:**
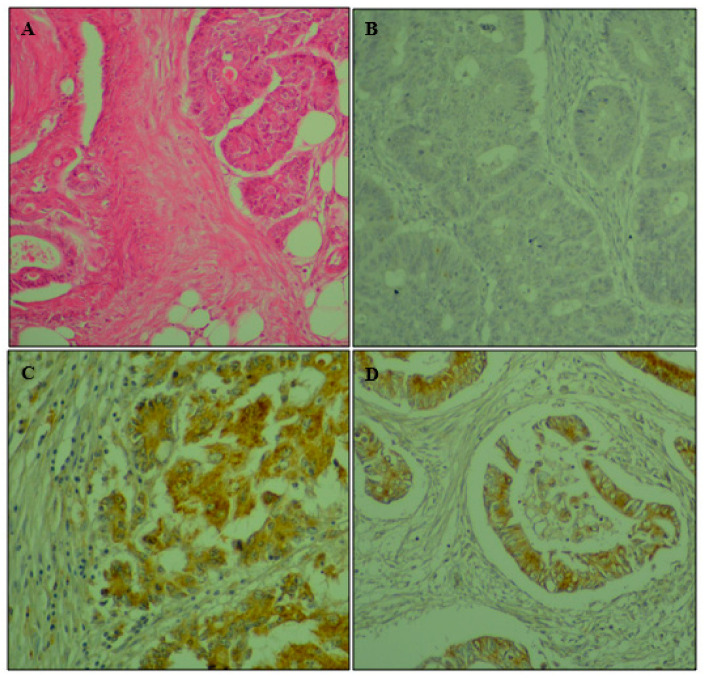
Immunohistochemical staining for cytoplasmic expression of ERCC1 in colorectal adenocarcinoma. (**A**) Hematoxylin and eosin × 400; (**B**) negative control × 400; (**C**) strong cytoplasmic staining for ERCC1; (**D**) moderate cytoplasmic staining for ERCC1× 400.

Status expression of ERCC1 in colorectal adenocarcinoma patients is presented in Table 2 and immunohistochemistry (IHC) staining in Figure 1.

**Figure 2:**
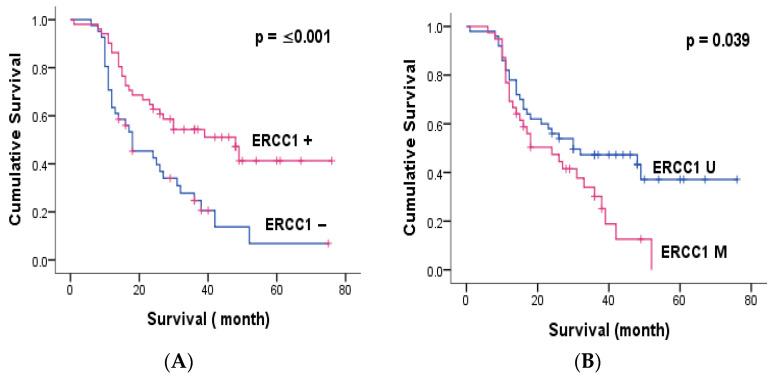
Kaplan–Meier overall survival curves of patients with colorectal cancer and either ERCC1 expression and methylation. (**A**) Kaplan–Meier curves indicating the effect of ERCC1 expression on patient survival, (**B**) Kaplan–Meier curves indicating the effect of ERCC1 methylation on patient survival.

Immunohistochemical analysis showed cytoplasmic expression of ERCC1 in 52.8% of colorectal adenocarcinoma patients included in the present study. Thus, low cytoplasmic expression was seen in 42.5%, while elevated levels were detected in only 10.4% of cases (Table 2). IHC analysis revealed that cytoplasmic ERCC1 staining was significantly and inversely related to distant metastasis (*p* = 0.038) and cancer recurrence (*p* = 0.05). Therefore, negative tumors were more likely to display distant metastasis and relapse. The expression of this protein was associated with a good clinical outcome in our study population (Table 3). Therefore, patients bearing a positive tumor had significantly greater 3-years OS and 5-years OS compared to negative ones (*p* = 0.002, *p* ≤ 0.001) (Table 6, Figure 2). 

### 3.3. ERCC1 and MGMT Methylation

**Figure 3:**
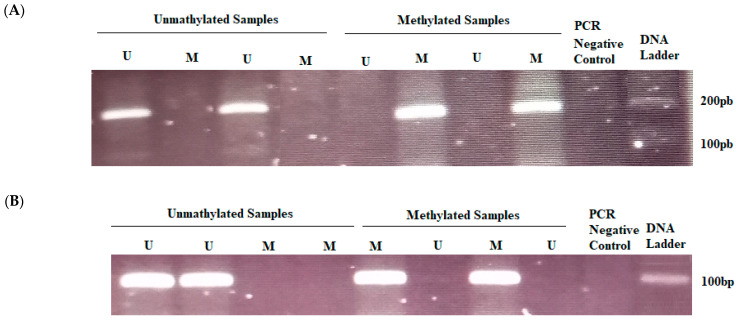
Agarose gel electrophoresis of methylation-specific PCR (MS-PCR) analysis of ERCC1 and MGMT. (**A**) Agarose gel electrophoresis of (MS-PCR) of ERCC1. (**B**) Agarose gel electrophoresis of (MS-PCR) of MGMT.

We detected the methylation profile of ERCC1 and MGMT using MSP (Figure 3). ERCC1 was methylated in 44.6% of colorectal adenocarcinoma while MGMT was methylated in 69% of cases (Table 2). No methylation was noted in non-malignant colorectal tissues for both genes. Therefore, a significant difference in ERCC1 and MGMT methylation between normal tissues and colorectal adenocarcinoma was reached (*p* ≤ 0.001). ERCC1 methylation was observed in 40% of early-stage cancers compared to 47% in the advanced cancer stages. In fact, no significant differences were noted in methylation levels according to staging, while it was significantly associated with well-differentiated tumors. Importantly, ERCC1 methylation was negatively associated with FOLFOX chemoresistance (*p* = 0.002). Methylation level was significantly higher in responder patients compared with non-responders (Table 3). 

MGMT methylation was detected in 59% in the advanced cancer stage compared to 41% in early stage. This difference was statistically significant (*p =* 0.022). Additionally, tumors bearing MGMT methylation had a significant trend toward lymph node metastasis (*p =* 0.001), lymph invasion (*p =* 0.005), venous invasion (*p =* 0.004), perineural invasion (*p* ≤ 0.001), distant metastasis (*p* ≤ 0.001) and relapse (*p* = 0.008). Additionally, MGMT methylation was significantly higher in chemosensitive patients than chemoresistant patients (*p=* 0.021) (Table 3). 

The simultaneous methylation of ERCC1 and MGMT was observed in 34% of patients. A correlation between combined promoter methylation of these genes and clinicopathologic parameters was founded. Hence, we found that this combination was strongly associated with the worst prognosis. There were higher methylation frequencies in patients with advanced clinical stage, differentiation, positive lymph node metastasis, venous invasion, perineural invasion, distant metastasis and recurrence. Importantly, tumors with a positive response were frequently methylated for both ERCC1 and MGMT (Table 4). 

Multivariate analysis binary logistic regression confirmed that simultaneous methylation of these repair genes was significantly and positively associated with perineural invasion (*p* = 0.048), distant metastasis (*p* ≤ 0.001) and cancer relapse (*p* = 0.018) (Table 5). 

**Figure 4:**
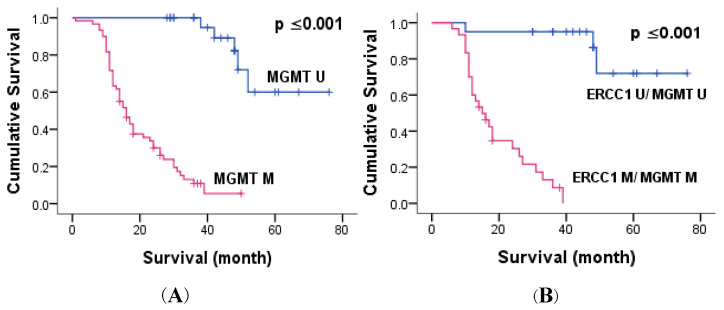
Kaplan–Meier overall survival curves of patients with colorectal cancer and either methylation of MGMT, alone or combined with ERCC1; (**A**) Kaplan–Meier curves indicating the effect of MGMT methylation on patient survival; (**B**) Kaplan–Meier curves indicating the effect of combined methylation of ERCC1 and MGMT on patient survival.

Kaplan–Meier survival analysis indicated that patients bearing a tumor with perineural invasion (*p* = 0.006, *p* = 0.002) and distant metastasis (*p* ≤ 0.001, *p* ≤ 0.001) had significantly reduced 3-year OS and 5-year OS compared to negative ones. Exploring the potential role of gene methylation, we found that the methylation of each gene (*p* ≤ 0.039, *p* ≤ 0.001 for ERCC1 and MGMT, respectively) and combined methylation (*p* ≤ 0.001) were significantly related to short 5-year OS (Figure 2 and Figure 4). Multivariate Cox proportional hazards model analysis confirmed that MGMT methylation was an independent predictive biomarker of short OS (*p* ≤ 0.001) (Table 6).

### 3.4. Correlation of ERCC1 Polymorphism with Clinicopathological Parameters, ERCC1 Expression and Methylation

Of the 37 patients, a predominance of the C/T genotype (51.4%) was noted as compared to C/C (32.4%) and T/T (16.2%) genotypes. This substitution was not correlated with most clinicopathological factors. Interestingly, the ERCC1 codon 118 C/T polymorphism was strongly associated with distant metastasis (*p* ≤ 0.001) and cancer resistance (*p* ≤ 0.001). Hence, T/T genotypes were significantly more observed in tumor-bearing distant metastasis. No correlation between this polymorphism, ERCC1 expression and methylation was found. Patient survival is significantly influenced by ERCC1 polymorphism (*p* = 0.031). Thus, the C/C genotype exhibited the best overall survival.

## 4. Discussion

In addition to their role in cancer prevention, ERCC1 and MGMT genes were considered as markers for the therapeutic response of many types of cancer, including colorectal cancer. Alterations in the expression of these genes can occur due to many mechanisms, including mutations and CpG promoter methylation. A marked increase in ERCC1 protein expression was observed in patients with distant metastasis (*p* = 0.039). The expression of ERCC1 had a significant impact on 3-OS and 5-OS. Our findings identified ERCC1 overexpression as a prognostic marker predicting longer impact on 3-OS and 5-OS. Consistent with our results, Jiang et al. showed that ERCC1 expression was significantly associated with reduced 3-year survival rates for CRC patients [8]. These findings were in accordance with those noted by the Egyptian report by Kassem et al. [18]. A recent study showed that tumors with wild-type KRAS and ERCC1 overexpression have significantly worse outcomes compared to negative tumors [19]. Other studies described ERCC1 as a prognostic biomarker in CRC [20,21]. 

Regarding the correlation of SNP with clinical outcome, our results indicated that patients harbouring a T/T genotype exhibited distant metastasis and short overall survival. In a recent study by Rao et al. C/C genotype presented the highest survival [10]. Our results were contrasted by the study of Gajjar et al., demonstrating no correlation between ERCC1 C118T polymorphism and clinicopathological factors [9]. The current study failed to find any correlation between ERCC1 C118T polymorphism and objective response to FOLFOX. Similarly, an Iranian study showed no significant association between response rate and genotypes [22]. Paradoxical findings were noted by a Chinese report. This confirmed a significant association of C/C genotype with survival time [23]. 

As our results indicated, about 55% of colorectal tumors showed methylation of ERCC1-promoter region, which is significantly higher than normal adjacent control. It was significantly and positively associated with cancer differentiation and poor 5-OS (*p* = 0.039). Shalaby et al. found that ERCC1 methylation was significantly higher in rectum cancer than in benign tumor while it was not correlated with any clinicopathologiclal factor [14]. 

Considering the methylation of MGMT, advanced-stage cancer specimens were found to have a significantly higher level of methylation compared to early stages and non-malignant CRC in the present study. MGMT methylation gradually increased in parallel with tumor progression and may serve as a biomarker for assessing CRC progression. We also showed that tumors bearing lymph node metastasis, distant spread, perineural invasion and cancer relapse are more likely to incur promoter methylation compared with negative ones. On the correlation of methylation with overall survival, 3-OS and 5-OS were significantly lower in patients harbouring MGMT methylation compared to negative ones. Loss of MGMT expression has a significant impact on various types of cancers, mainly colorectal cancer [16,24,25,26]. Hence, Ahmed et al. noted that MGMT expression was significantly correlated with tumor stage and metastatic status [16]. In accordance with our result, a further study showed that MGMT methylation was progressively increased during the normal–adenoma–carcinoma evolution [27]. Conflicting results demonstrated the absence of a correlation among MGMT methylation and overall survival [28]. According to Shalaby et al., MGMT methylation was not correlated with clinicopathological features while it was related to chemotherapy response in rectum cancer [14]. 

A combination of biomarkers may better reflect tumour aggressiveness and increase the clinical discriminative and prognostic value. Further, combining target molecules’ expression may be needed to adequately select patients who will benefit from chemotherapy. 

Based on current observations, tumors harbouring a combined methylation of MGMT and ERCC1 had a strong tendency towards lymph node metastasis, distant spread, perineural invasion, cancer relapse, and overall survival. These findings confirmed the critical role of these repair genes in advanced tumorigenesis steps. The promoter methylation of ERCC1 and MGMT is an essential event in cancer progression, spread, relapse, and could be clinically useful in assessing colorectal cancer progression and survival. 

ERCC1 and MGMT are known to be related to oxaliplatin- and alkylating-agent-based treatment [29,30]. Considering this, these repair enzymes might help predict FOLFOX resistance. Our results showed a strong association with chemotherapy response. Hence, the repair gene methylation, either alone or combined, was frequently observed in chemosensitive CRC patients. A recent study confirmed the correlation between MGMT-promoter methylation and response to alkylating agent-based treatment [31]. Pietrantonio et al. revealed that colorectal cancer patients with positive MGMT expression did not benefit from CAPTEM [32]. Several studies had already demonstrated that ERCC1 played a predictive role in FOLFOX-based chemotherapy resistance. Park et al. noted a correlation among ERCC1 overexpression and oxaliplatin regimen [19]. Further study failed to demonstrate any benefit in terms of efficacy [33]. Moreover, Rao et al. suggested that ERCC1 could serve as a marker of oxaliplatin response and the profile C/C or C/T genotype in ERCC1 rs11615 locus decreased benefit from oxaliplatin [10]. Our study showed an association between the simultaneous methylation of these genes and FOLFOX response. Further investigations are needed to determine the potential impact of these genes on chemotherapy response.

## 5. Conclusions

The current work confirmed a significant association between ERCC1 and MGMT methylation, either alone or combined with poor prognosis, relapse and low survival in colorectal cancer patients. Moreover, the methylation of repair genes could be a potential indicator of low chemosensitivity. However, our study is limited by the lower number of patients, which did not enable us to reach a conclusion about methylation in chemotherapy response. Further, larger studies are still needed to confirm these data.

## Figures and Tables

**Table 1:** Primer sequences and annealing temperature.

Primer		Sequence	Annealing Temperature
MSP ERCC1	UF	5′-TGGAATTGTTGGTGAGGGTTTTG-3′	55
	UR	5′-ACCTTCCCCTCCTCTCAACTT-3′	
	MF	5′-CGGAATTGTCGGTGAGGGTTTCG-3′	58.5
	MR	5′-ACCTTCCCCTCCTCTCAACTT-3′	
MSP MGMT	UF	5′-TTGTGTTTTGATGTTTGTAGGTTTTTGT-3′	55
	UR	5′-AACTCCAGACTCTTCCAAAAACAAAACA-3′	
	MF	5′-TTTCGACGTTCGTAGGTTTTCGC-3′	57
	MR	5′-GCACTCTTCCGAAAACGAAACG-3′	
ERCC1 SNP	F	5′-GCAGAGCTCACCTGAGGAAC-3′	56.5
	R	5′-GAGGTGCAAGAAGAGGTGGA-3′	

**Table 2:** Clinicopathological factors of colorectal cancer patients.

Clinicopathological parameters	**Factors**		**N**	**%**
	All cases		111	
	Gender	Men	65	41.4
		Women	46	58.6
	Age	≤50	35	31.5
		>50	76	68.5
	Anatomic site	Colon	96	86.5
		Rectum	15	13.5
	Differentiation	Well	75	67.6
		Moderate–poor	36	32.4
	Lymph node metastasis	No	45	40.5
		Yes	66	59.5
	Lymph invasion	No	39	35.1
		Yes	72	64.9
	Perineural invasion	No	55	49.5
		Yes	56	50.5
	Venous invasion	No	75	67.6
		Yes	36	32.4
	T Stage	T1–T2	58	53.7
		T3–T4	50	46.3
	Lymph node metastasis	No	45	40.5
		Yes	66	59.5
	Distant metastasis	No	45	45.5
		Yes	55	55.5
	Cancer relapse	No	19	31.7
		Yes	41	68.3
	FOLFOX	Non-responder	10	74.1
		Responder	17	25.9
IHC	ERCC1 expression	Negative	50	47.2
		Positive	56	52.8
	ERCC1 methylation	Negative	56	55.4
		Positive	45	44.6
	MGMT methylation	Negative	31	31
		Positive	69	69
	ERCC1 rs11615	CC	12	32.4
		CT	19	51.4
		TT	6	16.2

**Table 3:** Correlation between methylation and clinicopathlogical factors.

Factors	ERCC1 Expression		ERCC1 Methylation		MGMT Methylation	
	*r*	*p*	*r*	*p*	*r*	*p*
Gender	−0.080	0.417	0.059	0.558	0.057	0.575
Differentiation	−0.148	0.129	0.199	0.047 *	0.070	0.478
Lymph invasion	0.021	0.827	−0.112	0.276	0.282	0.005 *
Venous invasion	0.030	0.764	0.006	0.950	0.286	0.004 *
Perineural invasion	0.015	0.876	0.006	0.952	0.409	≤0.001 **
Stage	−0.056	0.574	0.005	0.965	0.232	0.022 *
Lymph node metastasis	0.052	0.594	0.079	0.436	0.322	0.001 *
Distant metastasis	−0.213	0.039 *	0.105	0.323	0.606	≤0.001 **
Cancer relapse	−0.234	0.074	0.196	0.156	0.356	0.008 *
Cancer resistance	0.054	0.688	−0.575	0.002 *	−0.420	0.021 *
ERCC1 expression			−0.700	≤0.001 **	0.255	0.013 *
MGMT methylation	−0.215	0.037 *	0.376	≤0.001 **		
ERCC1 methylation	−0.693	≤0.001 **				

Analysis using Spearman correlation; *r*: correlation coefficient; * *p* ≤ 0.05: significant; ** *p* ≤ 0.001: highly significant.

**Table 4:** Correlation between simultaneous methylation of ERCC1 and MGMT and clinicopathological factors.

Factors	Combined Methylation of MGMT and ERCC1	
	*R*	*p*
Differentiation	0.232	0.08 *
Lymph invasion	0.187	0.170
Venous invasion	0.283	0.036 *
Perineural invasion	0.417	0.002 *
Stage	0.283	0.036 *
Lymph node metastasis	0.244	0.07 *
Distant metastasis	0.605	≤0.001 **
Cancer relapse	0.446	0.014 *
Cancer resistance	0.655	0.006 *

Analysis using Spearman correlation; *r*: correlation coefficient; * *p* ≤ 0.05: significant; ** *p* ≤ 0.001: highly significant.

**Table 5:** Binary logistic regression analysis of the association between clinicopathological factors and simultaneous methylation of ERCC1 and MGMT.

Factor	Multivariate Analysis (*p*)
Stage	0.351
Differentiation	0.507
Lymph node metastasis	0.684
Lymph invasion	0.912
Venous invasion	0.124
Perineural invasion	0.048 *
Distant metastasis	≤0.001 **
Cancer relapse	0.018 *
Survival	≤0.001 **

* *p* ≤ 0.05: significant; ** *p* ≤ 0.001: highly significant.

**Table 6:** Survival analysis, clinicopathological factors and methylation in colorectal adenocarcinoma.

Factor	Univariate Analysis			Multivariate Analysis
	Overall Survival	3-Year OS	5-Year OS	
	*p*	*p*	*p*	*p*
Lymph node metastasis	0.210	0.333	0.210	0.362
Lymph invasion	0.207	0.235	0.207	0.9
Vascular invasion	0.691	0.8	0.691	-
Perineural invasion	0.003 *	0.008 *	0.003 *	0.444
Venous invasion	350	0.404	0.350	-
Distant metastasis	≤0.001 **	≤0.001 **	≤0.001 **	0.001 **
ERCC1 expression	≤0.001 **	0.002 *	≤0.001 **	0.05 *
ERCC1 methylation	0.039 *	0.062	0.039 *	0.276
MGMT methylation	≤0.001 **	≤0.001 **	≤0.001 **	≤0.001 **
Combined methylation	≤0.001 **	≤0.001 **	≤0.001 **	-

Survival analysis using Kaplan–Meier and; * *p* ≤ 0.05: significant; ** *p* ≤ 0.001: highly significant.

## Data Availability

Not applicable.
